# Evaluation of the public health impacts of traffic congestion: a health risk assessment

**DOI:** 10.1186/1476-069X-9-65

**Published:** 2010-10-27

**Authors:** Jonathan I Levy, Jonathan J Buonocore, Katherine von Stackelberg

**Affiliations:** 1 Department of Environmental Health, Boston University School of Public Health, Boston, MA, USA; 2 Department of Environmental Health, Harvard School of Public Health, Boston, MA, USA; 3 Harvard Center for Risk Analysis, Boston, MA, USA

## Abstract

**Background:**

Traffic congestion is a significant issue in urban areas in the United States and around the world. Previous analyses have estimated the economic costs of congestion, related to fuel and time wasted, but few have quantified the public health impacts or determined how these impacts compare in magnitude to the economic costs. Moreover, the relative magnitudes of economic and public health impacts of congestion would be expected to vary significantly across urban areas, as a function of road infrastructure, population density, and atmospheric conditions influencing pollutant formation, but this variability has not been explored.

**Methods:**

In this study, we evaluate the public health impacts of ambient exposures to fine particulate matter (PM_2.5_) concentrations associated with a business-as-usual scenario of predicted traffic congestion. We evaluate 83 individual urban areas using traffic demand models to estimate the degree of congestion in each area from 2000 to 2030. We link traffic volume and speed data with the MOBILE6 model to characterize emissions of PM_2.5 _and particle precursors attributable to congestion, and we use a source-receptor matrix to evaluate the impact of these emissions on ambient PM_2.5 _concentrations. Marginal concentration changes are related to a concentration-response function for mortality, with a value of statistical life approach used to monetize the impacts.

**Results:**

We estimate that the monetized value of PM_2.5_-related mortality attributable to congestion in these 83 cities in 2000 was approximately $31 billion (2007 dollars), as compared with a value of time and fuel wasted of $60 billion. In future years, the economic impacts grow (to over $100 billion in 2030) while the public health impacts decrease to $13 billion in 2020 before increasing to $17 billion in 2030, given increasing population and congestion but lower emissions per vehicle. Across cities and years, the public health impacts range from more than an order of magnitude less to in excess of the economic impacts.

**Conclusions:**

Our analyses indicate that the public health impacts of congestion may be significant enough in magnitude, at least in some urban areas, to be considered in future evaluations of the benefits of policies to mitigate congestion.

## Background

Congestion arises when a roadway system approaches vehicle capacity, resulting in numerous negative impacts ranging from wasted fuel and time to increases in tailpipe emissions. Multiple studies have modeled congestion in urban areas and assigned economic values to the excess fuel consumption and time wasted in traffic, concluding that congestion leads to annual economic burdens ranging from $83 billion [1] to $124 billion [2]. While this represents a substantial economic loss, there are multiple externalities from congestion that have not been previously characterized, including the public health impacts of excess air pollutant emissions during periods of congestion.

Fine particulate matter (PM_2.5_) is influenced by motor vehicle emissions of both PM_2.5 _and particle precursors, with source apportionment studies finding vehicles contributing up to one-third of ambient PM_2.5 _in urban areas in the US [3-5], with an even greater contribution if secondary sulfate and nitrate are considered. PM_2.5 _has been associated with premature mortality in multiple studies [6-8], and health impact assessments have demonstrated PM_2.5_-related damages on the order of hundreds of billions of dollars per year [9]. Recently, an expert committee [10] summarized the available epidemiological literature on exposure to traffic-generated air pollution and adverse health effects. They find strong evidence for a causative role for traffic-related air pollution on mortality, particularly from cardiovascular events. Thus, the public health implications of congestion could be appreciable and merit further investigation.

Multiple factors can complicate the comparison between economic and public health impacts, including some of the non-linearities in the system and variability across urban areas. On the former point, when density is close to capacity, small increases in traffic volumes generally lead to larger increases in delays (i.e., congestion follows a non-linear function) [11]. Economic impacts will tend to increase approximately proportional to delay time, but public health impacts will have somewhat different dependencies, including relationships with population size and age distribution (both of which will also influence traffic demand). On the latter point, previous studies [12,13] have emphasized that the population exposure implications of a given magnitude of emissions will vary significantly by location, largely as a function of population density at various distances from the source but also influenced by atmospheric conditions affecting pollutant fate and transport. Any analysis designing congestion mitigation strategies would need to take this variability into account, but complex chemistry-transport models are computationally intensive and may be impractical if direct economic costs dominate.

Yet another complication stems from the time-dynamic aspects of the system. While recent economic conditions have led to reductions in vehicle-miles traveled for passenger vehicles and goods transport, population and economic growth over the long term would be expected to lead to further increases in traffic volumes in upcoming decades. Without changes to the existing infrastructure (whether through increased highway construction, increased mass transit, or other solutions) or other policy interventions, substantially greater congestion would be anticipated, with corresponding economic and public health implications. However, there are countervailing influences, including significantly (presumably) lower per-vehicle emissions of multiple pollutants over time.

Thus, the primary aims of our study are to estimate the magnitude of the air pollution-related public health impacts of congestion relative to the economic impacts of congestion, in order to determine the significance of public health endpoints in future assessments and to identify information gaps that need to be addressed in order to accurately determine the future burden of congestion in the United States and to better evaluate potential strategies for ameliorating congestion. Our study considers a "business as usual" scenario with the following objectives:

• Develop a baseline scenario to predict time spent in congested traffic in 83 urban areas out to 2030

• Quantify emissions of PM_2.5 _and particle precursors as a function of vehicle speed, urban area, and year

• Develop estimates of health risks and monetary impacts attributable to congestion, using a value of a statistical life approach

• Determine the critical information gaps that must be addressed in order to more precisely quantify the public health burdens of congestion and the public health benefits of potential congestion mitigation measures.

## Methods

The key components of the analysis include predicting emissions corresponding with traffic congestion for 83 individual urban areas based on travel demand models, developing estimates of changes in ambient concentrations associated with these emissions, applying concentration-response functions for the contaminants of concern, and finally, integrating the components of the model to estimate potential health risks associated with exposure to pollutants attributable to congestion. We focus on primary and secondary PM_2.5 _as the constituents of concern, and evaluate only premature mortality attributable to PM_2.5 _exposures, noting that there are numerous morbidity effects including respiratory and cardiovascular outcomes that are not considered in this analysis.

We note that there are two primary exposures potentially resulting from emissions during congestion events: the first is in-cabin exposures for drivers in their vehicles, and the second is a general increase in ambient concentrations of contaminants that impact the surrounding population. In the present study we focus solely on quantifying impacts associated with increases in ambient concentrations.

### Predicting Emissions

We develop estimates of vehicle miles traveled (VMT) based on data and methods from the Center for Urban Transportation Research (CUTR) at the University of Central Florida [14]. We use MOBILE6 to estimate city-specific emissions per VMT based on year, temperature profile, and average vehicle speed. We focus on emissions from the baseline year (2000) until 2030. The analysis is conducted for 83 individual urban areas that were previously evaluated by the Texas Transportation Institute (TTI) [1] and are in the lower 48 states. The following sections provide more detail concerning each of these analytical steps.

#### Vehicle Miles Traveled (VMT) Estimation

We obtained census data and projections for different age classes from Woods & Poole [15], for each county of the United States, for the years 2000 - 2015, 2020, 2025, and 2030. To properly apply the CUTR model, we first determined the population in each of the 83 urban areas modeled (Additional file 1, Table S1). To approximate urban area population, we began by establishing baseline population data using 2000 US Census data at census block resolution, overlaying these data on shapefiles for the urban areas of interest. This provided estimates not only of the population within each urban area as a whole, but also estimates split by county when the urban area spanned multiple counties.

To determine urban area population for past and future years, we calculated the percentage change in population for each county relative to 2000 using Woods & Poole data, and we assumed that these percentages were applicable to the portions of the urban areas located within each county.

Next, predictions of traffic volume were based on a model derived from an analysis of the National Household Travel Survey, part of the 2000 US Census, developed by Polzin and Chu at CUTR [14] in a spreadsheet model they made available. The CUTR model inputs include age distribution, population density, gender distribution, and residency tenure distribution as covariates. We estimated population age distribution using Woods & Poole data, we calculated population density directly from the population estimates, and residency tenure distributions were provided in the CUTR model on a state-by-state basis for 2001 and 2035, and were linearly interpolated to provide values for the intervening years. These age, population density, and residency tenure covariates, represented as proportion of the population, were multiplied by factors determined by the CUTR analysis to estimate the different factors of travel behavior - person-trips/person, person-miles/person-trip, and vehicle-miles/person-mile.

(1) $$\begin{array}{l} \frac{\text{Person}\text{​}\text{-}\text{​}\text{trips}}{\text{Person}}=\text{State }\;\text{constant}-\\ \sum \begin{array}{l} {}[\text{proportion }\;\text{in}\;\text{age }\;\text{group}\times \\ \text{multiplier}\;\text{for }\;\text{age }\;\text{group} \end{array}]-\\ \sum \begin{array}{l} {}[\text{proportion }\;\text{in}\;\text{residency }\;\text{tenure }\;\text{group}\times \\ \text{residency }\;\text{tenure }\;\text{group }\;\text{multiplier}] \end{array}-\\ \sum \begin{array}{l} {}[\text{proportion }\;\text{in population }\;\text{density }\;\text{group }\times \;\\ \text{population }\;\text{residency }\;\text{multiplier}] \end{array} \end{array}$$

Person-miles/person-trip and vehicle-miles/person-mile were calculated similarly, and the product of these three terms and population provided VMT, as indicated below.

(2) $$\begin{array}{l} \text{VMT}=\text{population}\times \frac{\text{person}\;\text{trips}}{\text{person}}\times \\ \frac{\text{person}\;\text{miles}}{\text{person}\;\text{trips}}\times \frac{\text{vehicle}\;\text{miles}}{\text{person}\;\text{miles}} \end{array}$$

We estimated VMT on a per-county basis using the fraction of the population of that county actually residing in the urban area, then summed across the entire urban area to generate estimates applicable for that urban area. The VMT estimates then fed into the travel demand and congestion model developed by TTI. We note that the TTI model links VMT with congestion but does not include forecasted VMT, necessitating the use of two different models, but that this leads to some incompatibilities (e.g., the CUTR model is driven by population within the urban area, whereas the historical TTI analyses are driven by traffic volume data that includes people not residing in the urban area). To evaluate our approach before proceeding with the core analyses, we compared our VMT estimates to those from TTI for past years (1985, 1990, 1995, and 2000 - 2005), while recognizing that the models would not be expected to yield identical outputs given these differing assumptions between the TTI and CUTR model inputs.

#### Travel Demand and Congestion Modeling

We combined the VMT estimates derived above with population data at the census tract level for the 83 urban areas addressed by TTI. The household travel survey provides the data which CUTR used to construct the traffic demand function. This is difficult to estimate, as the data do not address induced travel resulting from increased roadway capacity [16-18]. The baseline scenario presented here assumes the demand elasticity for trip rate, trip length, and occupancy derived from analyses of the CUTR surveys. Demand elasticities are primarily related to fuel price, travel time, and income [19].

Estimates of the infrastructure in each urban area were provided by TTI. Data were only provided for years between 1985 and 2005, and we used values for 2005 for all subsequent years (e.g., a fixed infrastructure over time). The values for VMT in each urban area were divided by the available infrastructure to generate daily traffic per lane. This fed into equations used to estimate average vehicle speed on freeway and arterial streets, and are based on uncongested to extremely congested conditions [1].

The percent of daily travel under congested conditions was based on the roadway congestion index, estimated as the ratio of daily traffic volume to the number of lane-miles of arterial streets and freeways. TTI [1] provides a non-linear function relating roadway congestion index to the amount of travel occurring in congested conditions (Additional file 1, Table S2) that imposes a maximum of 50% of daily traffic occurring in congestion. Using traffic volume and infrastructure estimates, the average speed on both arterial streets and freeways in both peak and off-peak directions can be estimated using the equations provided by TTI (Additional file 1, Table S3). The split of traffic between peak and off-peak directions was assumed at 65% and 35%, respectively, in accordance with median values reported previously [20].

#### MOBILE6

Emissions are estimated using the MOBILE6 vehicle emission modeling software from the US EPA [21], the most robust software available at the time of our analysis. Given interests in PM_2.5_-related health risks, we derived emissions estimates for nitrogen oxides (NOx), sulfur dioxide (SO_2_), and primary PM_2.5 _for all model years, based on monthly averages of daily maximum and minimum ambient temperature, average vehicle speed for the two road types from the speed model, and MOBILE6 default fleet composition and performance for that year. A key limitation of MOBILE6 is that emissions are estimated using an average speed; thus, significant aspects of stop and go traffic and fast acceleration and deceleration, hallmarks of travel in congestion, are not adequately captured. Because of this limitation, within our study, we modeled the emissions that occur during congested conditions (with 100% of the emissions during periods of congestion attributed to "congestion"), rather than evaluating the proportion of emissions due solely to reduced vehicle speeds. In other words, the emissions outputs from MOBILE6 for each urban area were multiplied by the amount of VMT that occurs in congestion. This provided an estimate of the health risks associated with periods of congestion, but not information on (for example) the marginal difference between current conditions and an enhanced infrastructure that would allow for the same traffic volume at higher speeds, which was beyond the capabilities of MOBILE6 and therefore outside of the scope of our study.

### Exposure Estimates

To estimate the marginal concentration changes associated with congestion-related emissions from each urban area, we applied a source-receptor (S-R) matrix [22,23]. S-R matrix is a reduced-form model containing county-to-county transfer factors across the United States, considering both primary PM_2.5 _and secondary formation of sulfate and nitrate particles. It is based on an underlying sector-averaged Gaussian dispersion model with wet and dry deposition and first-order chemical conversion. The S-R matrix is simplified relative to gold standard chemistry-transport models such as the Community Multiscale Air Quality model (CMAQ), but it is computationally tractable for an application such as this, and it has been shown to yield similar population health impact estimates to CMAQ [24,25] and CALPUFF [26] at a fraction of the computational time and cost. It also includes a calibration step to ensure correspondence with ambient monitoring data. The calibration factors were developed comparing the modeled PM_2.5 _concentrations at county centroids with spatially interpolated monitored data at county centroids. For the monitored data, 2001 National Emissions Inventory (NEI) and data from the Federal Reference Method (FRM) and EPA's Speciation Network (ESPN) monitor sites were used.

### Concentration-Response Function for PM_2.5 _Mortality

PM_2.5 _has been associated with a number of morbidity outcomes as well as premature mortality. For the purpose of this assessment, we focus on mortality due to long-term exposure to PM_2.5_, which has previously dominated monetized externality estimates in comparison with morbidity endpoints [9,27]. As in recent health impact assessments [28], we derive our concentration-response function from a combination of published cohort studies and an expert elicitation study addressing the concentration-response function for PM_2.5_-related mortality. Two major cohort studies are generally thought to provide estimates that are most robust and applicable to the general population, with the Harvard Six Cities Study publications reporting central estimates of an approximate 1.2-1.6% increase in all-cause mortality per μg/m^3 ^increase in annual average PM_2.5 _[7,8], and the American Cancer Society studies reporting estimates of approximately 0.4-0.6% [6,29], with higher estimates when exposure characterization was more spatially refined [30]. Within the expert elicitation study [31], the median concentration-response function across experts was approximately 1%, midway between these cohort estimates, with a median 5^th ^percentile of 0.3% and a median 95^th ^percentile of 2.0%. For this first-order health impact calculation, we consider a value of a 1% increase in all-cause mortality per μg/m^3 ^increase in annual average PM_2.5 _to be well-justified and applicable, but consider the implications of alternative values in sensitivity analyses. We applied this function to the baseline mortality rate and the number of people in each census tract 25 years of age or older (the population in the Six Cities Study). We assumed that the age-specific mortality rate would not change over time, but given shifts in the age distribution of the population, that the overall mortality rate could change over time.

### Monetized Estimates of Premature Mortality

To monetize the resulting estimates of mortality attributable to congestion, we applied a value of a statistical life (VSL) of approximately $7.7 M in 2007 dollars (for 2000 GDP), the central estimate used in recent EPA regulatory impact analyses [32]. We increased VSL as a function of predicted increase in real GDP (as reported by the Bureau of Labor Statistics) and an income elasticity of 0.5, noting that recent epidemiological evidence indicates that the time lag between exposure to PM_2.5 _and mortality is relatively short [8], indicating that our value would not be sensitive to choice or application of discount rate.

### Comparisons between Public Health and Economic Impacts

One of our primary objectives is to compare the monetized public health damages with the economic damages associated with congestion, over time and across urban areas. Although economic damages from fuel and time wasted have been derived previously, we re-estimated these values to correspond with our VMT estimates and to provide consistency with our public health estimates. We applied algorithms from TTI [1,14] to calculate time and fuel spent in traffic at the modeled speeds and at free-flow. We calculated the difference between the time and fuel consumed at modeled speeds and at free-flow, which gave us the time and fuel wasted as a result of time spent in congestion, allowing us to compare economic and public health costs from each of these sources.

While the structure of our analyses did not allow for formal uncertainty propagation, given the numerous parameters with no plausible uncertainty quantification, we recognize that the uncertainties in our monetized public health damage estimates are significant. In our core results, we present public health damages based on central estimates for each parameter to provide a first approximation of impacts, and within limited sensitivity analyses, focus on the influence of selected parameter values on qualitative conclusions regarding the relative magnitudes of public health and economic damages.

## Results

### Vehicle-Miles Traveled and Degree of Congestion

In total, across the 83 urban areas modeled, VMT is projected to increase 33% from 2000 to 2030 (an increase from 2.97 billion daily VMT to 3.94 billion daily VMT), closely paralleling projected population growth in the urban areas of 32% (an increase from 133 million people to 176 million). There are some clear geographic patterns in the areas with high and low percentage changes in VMT. Multiple urban areas in the South and West are projected to have VMT growth in excess of 40%, including Raleigh NC, Oxnard CA, Richmond VA, and San Antonio TX. Those urban areas with limited VMT growth or declines are generally found in the industrial Midwest, including Cleveland OH, Dayton OH, Detroit MI, and Toledo OH-MI, but also in New Orleans LA and Salem OR. Intermediate values are generally seen in the largest urban areas, with 7-10% projected growth in New York-Newark, Los Angeles, and Chicago. A complete table of VMT estimates by urban area is provided in Additional file 1, Table S4.

Trajectories of the degree of congestion by urban area closely pattern trajectories of VMT, although not in a linear fashion given the maximum value of 50% of time in congestion in the traffic model and other non-linearities in the system. Whereas 18 of the 83 urban areas were estimated to have 50% of time in congestion in 2000, 40 urban areas reached this threshold by 2030, given our baseline scenario of population growth with no corresponding changes in infrastructure or policy.

### Attributable Emissions and Public Health Impacts

**Figure 1:**
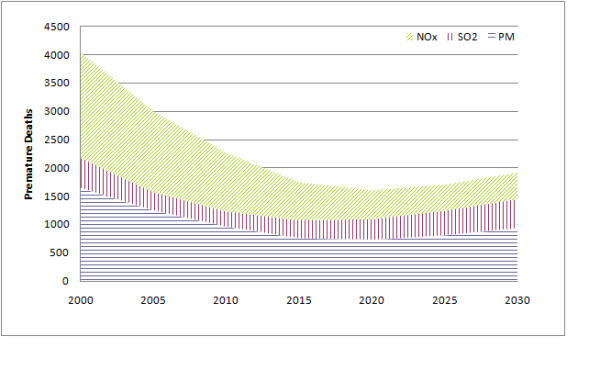
**PM_2.5_-related premature mortality attributable to traffic congestion nationwide. Pollutants in legend indicate emitted pollutants, and the figure indicates the effects of these emissions on PM_2.5 _concentrations**.

For 2005, nationwide estimates of traffic emissions attributable to time spent in congestion include approximately 1.2 million tons of NOx, 34,000 tons of SO_2_, and 23,000 tons of PM_2.5_. These emissions are associated with approximately 3,000 premature deaths in 2005 (Figure 1), with an economic valuation of $24 billion (in 2007 dollars). Overall, approximately 48% of the impact over the 83 urban areas is attributable to NOx emissions, with 42% attributable to primary PM_2.5 _emissions and 11% attributable to SO_2 _emissions. However, the relative proportion of the impact attributable to different pollutants varies significantly across urban areas. For example, the proportion due to NOx ranges from 6% in multiple Northeast cities (Hartford, CT; Boston, MA; New Haven, CT; Springfield, MA) to over 70% in less densely populated areas of Texas (Brownsville, Austin) and Washington State (Spokane). Similarly, the proportion due to primary PM_2.5 _emissions is highest in densely-populated urban areas of the Northeast (approximately 80%) and below 20% in Brownsville. The proportion attributable to SO_2 _emissions is highest in California, with four urban areas in California constituting the only places with more than 20% of the mortality risk from SO_2 _emissions. These relative proportions are attributable in part to high ambient sulfate in the eastern US, which tends to reduce particulate nitrate formation, and to atmospheric conditions in California favoring the secondary formation of particulate sulfates.

Examining time trends in mortality risk attributable to congestion (Figure 1), there is a steady decline from 2000 until 2020, with an estimated 4,000 premature deaths in 2000 and an estimated 1,600 premature deaths in 2020. This decline is driven by significant reductions in projected per-vehicle emissions that offset the growth in population and general increase in per-capita mortality rates (based on the aging of the population). However, after this point, there is a steady increase in mortality risk (to 1,900 premature deaths in 2030), as population growth continues but projected pre-vehicle emissions reductions level off. Using economic values assigned to premature mortality in each year, with all values in 2007 dollars, the impact decreases to approximately $13 billion in 2020, after which point it increases up to $17 billion by 2030. Changes occur in the relative contribution of various particle constituents, given that NOx emissions per vehicle-mile are projected to decrease by 83% on arterial streets and 86% on freeways between 2005 and 2030, as opposed to a 64% decrease in primary PM_2.5 _emissions on both road types and a 24% decrease in SO_2 _emissions on both road types. By 2030, the relative contributions of the different pollutants to mortality risk attributable to congestion have changed to 24% attributable to NOx emissions, 27% attributable to SO_2 _emissions, and 49% attributable to primary PM_2.5 _emissions.

**Figure 2:**
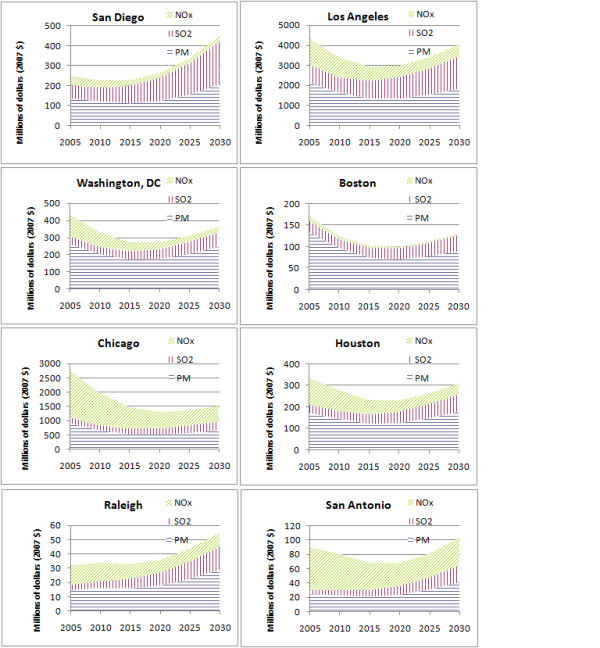
**Monetized estimates of the PM_2.5_-related mortality risks from traffic congestion in selected urban areas. **Pollutants in legend indicate emitted pollutants, and the figure indicates the effects of these emissions on PM_2.5 _concentrations.

Figure 2 presents the monetized health impacts over time for selected urban areas, illustrating that the trajectories differ as a function of differential population growth, congestion, population density and atmospheric chemistry. For example, monetized health impacts increase steadily over time in cities such as Raleigh NC and San Diego CA, in which VMT and population growth are significant and primary PM_2.5 _makes a substantial contribution to health risk. In contrast, Chicago and other cities in the Midwest are projected to have small VMT growth and have more substantial contributions to public health damages from NOx emissions, and therefore show a steady decline in health risks over time given the larger decline in NOx emissions per vehicle-mile.

### Comparison of Economic Impacts and Public Health Impacts

**Figure 3:**
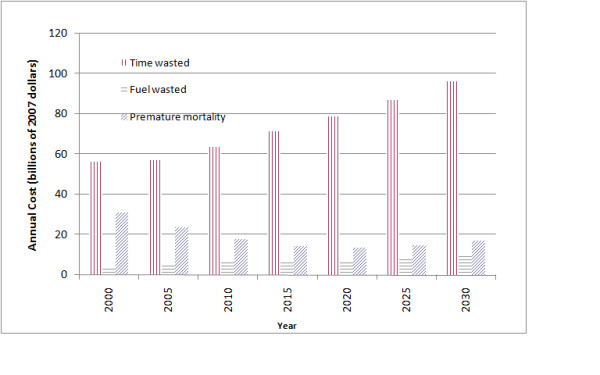
**Comparison of the economic costs of congestion with the monetized estimates of PM_2.5_-related mortality risks (in billions of 2007 dollars)**.

Figure 3 presents the economic costs from time and fuel wasted and monetized estimates of premature mortality attributable to traffic congestion across the 83 urban areas. Overall, time wasted accounts for the bulk of the economic cost associated with traffic congestion, and the cost of time wasted increases from $56 billion in 2000 to $96 billion in 2030, as this is directly proportional to the extent of congestion. In contrast, reductions in per-vehicle emissions contribute to declines in economic costs associated with premature mortality from $31 billion in 2000 to $13 billion in 2020, with modest increases after that point to $17 billion in 2030.

As a result, whereas the public health impacts contributed approximately 34% of the total cost of congestion in 2000, this decreases to 14% by 2030. However, the proportion of the cost of congestion attributable to premature mortality varies substantially across urban areas. For example, in 2000, 17 urban areas had health impacts contributing less than 20% of the total cost of congestion, whereas 19 urban areas had contributions in excess of 50%. Those urban areas with relatively small contributions from public health had very high levels of congestion (near or at the 50% threshold) but did not have correspondingly high population density, including Laredo TX, Eugene OR, and Las Vegas NV. In contrast, those urban areas where public health impacts dominated had smaller percentage of time spent in congestion but greater public health benefits per ton of emissions.

### Sensitivity Analyses

Our findings should be considered quite uncertain and are clearly sensitive to a number of the model assumptions, many of which could not be formally quantified or propagated. However, we note that the relative economic values assigned to the cost of fuel, the value of time per hour, and the value of statistical life, as well as future fuel efficiency, are key assumptions that could greatly influence our conclusions. In addition, we note that the magnitude of the public health damages is sensitive to the concentration-response function applied. Because our model results scale linearly with these values, we can provide some first-order approximations of the relative values that would be needed to alter our conclusions about the significance of public health relative to economic impacts, providing a sense of the likelihood of significantly different conclusions by year.

For example, in 2000, the price of gasoline is known, so the estimate of the cost of fuel wasted is relatively less uncertain. The cost of delay depends on the value of time, which we derived from TTI models using a non-commercial value of time (approximately $16/hr in year 2000 dollars), which is likely a lower bound on the true value of time. The VSL and the concentration-response function represent the most significant quantifiable uncertainties for public health damages, so we can explore the combinations of values needed for public health damages to exceed economic damages. Using the central estimate for the concentration-response function, the VSL would need to be approximately $14 million in 2007 dollars for the public health damages to exceed the value of time wasted in 2000, or roughly the 95^th ^percentile of the uncertainty distribution for VSL reported by US EPA [32]. Using an upper-bound concentration-response function of 2% would lead the public health damages with a central estimate VSL to be comparable to the economic damages, whereas a lower-bound concentration-response function of 0.3% would require an extremely large VSL for the magnitudes to be comparable. By 2005, the VSL would need to have been $18.5 million in 2007 dollars for public health damages to exceed the value of time using the central estimate concentration-response function, which is outside of the reported confidence intervals. When considering future years, uncertainties exist regarding both the value of time and the cost of fuel, with potentially large uncertainties in the latter case. However, across all model years, the cost of fuel contributes an order of magnitude less than the value of time, indicating that the cost of fuel would need to increase appreciably relative to income in order to strongly influence our conclusions (and such a change in the cost of fuel would appreciably influence driving patterns and resulting congestion). As the contribution from public health damages decreases relative to the value of time in future years, the necessary VSL and concentration-response function to equalize these damage estimates becomes quite high, although significant uncertainties regarding the time trajectory of emissions and effect of changing background concentrations on pollutant formation add to uncertainties in this comparison, and public health damages are the dominant contributors in selected urban areas even in 2030.

## Discussion

Our modeling illustrates that the public health impacts of traffic during periods of congestion, associated with premature mortality from primary and secondary PM_2.5 _concentrations, are appreciable, with thousands of deaths per year and a monetized value of tens of billions of dollars per year. While the monetized public health damages are smaller than the economic value of time wasted, with the differential anticipated to grow over time, there are some geographic areas where public health damages represent a significant proportion of the total damages, even in future years when per-vehicle emissions are expected to be substantially less. Prior analyses of population exposure per unit emissions from motor vehicles [13] demonstrated that these values were highest in dense urban areas for primary PM_2.5 _and secondary sulfate, especially in California, the mid-Atlantic states, and the industrial Midwest, and were highest in the Southeast and Midwest for secondary nitrate. The urban areas with the greatest proportion of damages from public health were often found in parts of California and the Midwest, where the damages per ton of emissions were greater and the projected future population growth was lower. These findings provide an indication that considering only the direct economic costs of congestion will underestimate societal benefits of mitigating congestion, significantly so in certain urban areas.

There are some clear limitations in our models and their interpretation, which provide some potential guidance for future studies. First, our results assume that the infrastructure is fixed at 2005 levels but that population continues to increase out to 2030. This is relatively unlikely to be the case, but given our modeling framework and available information, it is difficult to evaluate future changes in infrastructure in a manner that is not misleading. Increasing the available infrastructure (whether through roadway capacity or public transportation) increases average speed. Because of limitations in the MOBILE6 model, which assumes an average speed and does not incorporate stop-and-go traffic or fast acceleration and deceleration, increased speed leads to higher MOBILE6 emissions estimates, particularly for NOx. In contrast, Frey et al. [33] illustrated that when comparing traveling conditions of congestion and free-flowing traffic in which the estimated average speed is similar, emissions during congested driving conditions are 50% higher. Other studies have shown that NOx emissions from heavy-duty diesel vehicles are three times higher at low speeds than when at highway speed [34], and PM, elemental carbon (EC) and organic carbon (OC), and air toxics emissions are heightened during start-and-stop traffic [35,36], but these insights are not captured in our emissions model.

We therefore could only characterize the public health impacts of congestion as the total impacts during periods of congestion, rather than the marginal difference between these impacts and the impacts if the infrastructure were expanded, a potentially significant limitation. Future studies should explore other emissions modeling frameworks that better capture the effects of congestion on emissions, linked to more detailed traffic flow models. For example, micro-simulation techniques have been developed to evaluate the impact of improved traffic flow on vehicle emissions [37], capturing both the near-term decrease in emissions and the long-term effect of induced vehicle traffic. However, this model does not capture all pollutants or provide insight about the effects of changes in technology over time. The MOVES2010 emissions model [38] became available subsequent to our analysis, which provides some insight regarding start-and-stop traffic, and this modeling framework could be explored in future studies.

There are further limitations with MOBILE6 that should be acknowledged, including difficulties in addressing malfunctioning vehicles and in projecting fleet composition over an extended period of time. For comparability with other work and lacking other quantitative values, we used default MOBILE6 values where possible, including for fleet mixtures (by age and class), mileage accumulation, inspection and maintenance programs, vehicle fuel economy and emissions performance, starting emissions, reformulated gasoline and oxygenated fuels programs, and low-sulfur diesel regulations, among others. To the extent that there are significant regulatory or technology changes over time, which would influence per-vehicle emissions as well as fleet composition, our analyses would become progressively more uncertain over time. In an extreme case, given significant penetration of electric vehicles (which were not formally considered in this analysis), the impact of congestion would need to consider emissions from electricity production, which was well beyond the scope of this analysis. Regardless, this emphasizes that there are some large uncertainties in emissions characterization, which could not be quantified in our sensitivity analyses but which should be considered in formulating policy strategies.

A related limitation has to do with a difference in how congestion is treated within the economic and public health impact models. Our estimates of time and fuel wasted are derived from the difference between modeled speed and free-flow speed on arterial roads and freeways (assumed to be the posted speed limit), while the health impact estimates are based on the roadway congestion index, a piecewise linear function providing an estimate of the proportion of the daily traffic that occurs in congestion, which we then use to attribute emissions to congestion. Because changes in speed are only predicted once a threshold relative congestion index is reached, time and fuel wasted therefore have a congestion threshold below which there are no predicted impacts, although the roadway congestion index predicts emissions attributable to congestion at all levels. As a result, some areas will show public health impacts but little to no economic damages from wasted time and fuel. These situations should be interpreted as largely artifacts of the modeling structure, and a more refined characterization of traffic flows and infrastructure-based congestion would likely eliminate these predictions in future assessments. Additionally, the congestion model treats the entire urban area as a whole, so the model does not explicitly distinguish between arterial and freeway traffic conditions the way that the speed model does. Both this issue and the previous issue regarding infrastructure emphasize the potential need for more refined assessments in targeted geographic areas, in which modeling can go beyond what is feasible when looking at 83 urban areas concurrently. That said, modeling 83 urban areas concurrently provides first-order insight about geographic areas necessitating further study, allowing for targeted future studies.

An additional limitation arises in our focus solely on the mortality risks of PM_2.5_. There are clearly numerous other health endpoints or pollutants that may contribute to the public health burden of congestion, including morbidity endpoints associated with PM_2.5_, mortality and morbidity from ozone, and effects of multiple air toxics. As described earlier, previous studies have demonstrated that PM_2.5_-related mortality dominates monetized public health damages, but if lower concentration-response functions are applied for mortality or lower economic values are applied to premature mortality (e.g., using a life-year approach), the significance of PM_2.5_-related morbidity would be enhanced. In addition, in years and urban areas where the contributions from NOx emissions are large relative to the contributions from SO_2 _or primary PM_2.5 _emissions, inclusion of ozone concentrations may be warranted. We also omitted potential impacts of ultrafine particulate matter, which would be challenging to quantify given limitations with emissions inventories, dispersion models, and available concentration-response functions, but which could exhibit significant health effects in the near field and may merit future consideration.

One additional pollution-related endpoint of interest would be carbon dioxide emissions and corresponding effects of global climate change. While estimating this pathway is highly uncertain and was beyond the scope of our analysis, we can make a first-order approximation to determine the likelihood that this would contribute appreciably to damages. In 2005, we estimated approximately 3.12 billion daily VMT across the 83 urban areas during periods of congestion. According to a recent report [39], gasoline contributes climate-related damages on the order of 0.06 to 6 cents/VMT, which would correspond with between $680 million and $68 billion in damages, bounding the PM-related public health damages of $24 billion in 2005. While both categories are quite uncertain, this indicates that modeling global climate impacts clearly merits further consideration.

In addition, while we have included the effects of emissions on ambient concentrations, we have not captured the effects on in-vehicle concentrations and personal exposures. As drivers and passengers spend more time in traffic, their in-vehicle exposures to traffic-related pollutants will increase due to time-activity changes as well as increases in roadway concentrations, which can sharply increase personal exposures and doses. For example, Alm et al. [40] found that added exposures (due to commuting in addition to background levels) to carbon monoxide and PM_2.5 _were considerably higher during morning commutes and higher under slower average speed conditions than in faster driving conditions. Riediker et al. [41] measured PM and volatile organic compounds in vehicles at various times and found that elevated contaminant levels were related to locations with high traffic volumes. These and other studies suggest that in-vehicle exposures may significantly contribute to personal exposures, particularly during times of congestion, and these would add to the estimates of health impacts developed here. We could not quantify this pathway, given challenges in linking epidemiological evidence from central site monitors with personal exposures during commuting, as well as because of complications related to differences between ambient and in-vehicle concentrations, but we anticipate that this pathway would be appreciable enough to merit consideration in future analyses.

Although these and other factors are significant limitations, it is important to note that more advanced modeling approaches would be unable to characterize 83 urban areas individually and simultaneously, and would therefore lack insight on relative differences among urban areas. Moreover, our VMT estimates and resulting economic costs of congestion are quite similar to those previously determined [1], and our estimated public health damages per ton of emissions compare quite favorably to those estimated previously in spite of the simplified atmospheric model applied. For example, a recent study [42] used a response surface model derived from CMAQ runs to determine health impact estimates for nine urban areas in 2015. The estimated damage values for mobile sources were $550,000 per ton of primary PM_2.5 _and $9,700 per ton of NOx, with $73,000 per ton of SO_2 _for area sources (no value was reported for mobile sources). The corresponding values from our study (for all 83 urban areas) for 2015 are approximately $530,000 per ton of primary PM_2.5_, $11,600 per ton of NOx, and $100,000 per ton of SO_2_.

More generally, though capturing the congestion-related contribution from traffic emissions was challenging given available models, this framework was necessary to provide direct comparisons with the economic impacts of congestion. This would also allow for future evaluations of policy measures addressing congestion, including both infrastructure enhancements and other measures to reduce congestion (e.g., congestion pricing and traffic management). Thus, despite the limitations and somewhat simplified modeling approaches in our study, our findings can be used to inform both public policy and future investigations of the economic consequences of traffic congestion.

## Conclusions

Our model results show that health impacts (based on estimates of premature mortality following exposure to primary and secondary PM_2.5_) are likely significant enough to necessitate inclusion in a comprehensive evaluation of the benefits of measures to reduce congestion. While health impacts are projected to decrease relative to economic impacts, given reductions in per-vehicle emission rates, there remain multiple urban areas in which the public health impacts are still appreciable in 2030. We recommend that future investigations develop refined estimates of emissions in congestion and linkages with personal exposures, which could make a significant contribution to the estimated public health burdens of congestion in multiple urban areas.

## List of abbreviations

CMAQ: Community Multiscale Air Quality model; CUTR: Center for Urban Transportation Research; EC: elemental carbon; EPA: Environmental Protection Agency; ESPN: EPA Speciation Network; FRM: Federal Reference Method; GDP: gross domestic product; NEI: National Emissions Inventory; NOx: nitrogen oxides; OC: organic carbon; PM_2.5_: fine particulate matter; S-R: source-receptor; SO_2_: sulfur dioxide; TTI: Texas Transportation Institute; US: United States; VMT: vehicle miles traveled; VSL: value of statistical life

## Competing interests

The authors declare that they have no competing interests.

## Authors' contributions

JIL participated in study design, contributed to the concentration modeling and health impact estimates, and helped to draft the manuscript. JJB carried out the modeling of traffic, congestion, and emissions and led all analytical efforts. KVS conceived of the study, participated in its design and coordination, and helped to draft the manuscript. All authors read and approved the final manuscript.

## Supplementary Material

Additional file 1 **Supplemental information**. The supplemental information file includes a list of the 83 urban areas included in the model (Table S1), methods for estimating the percent of travel time in congestion (Table S2), estimates of vehicle speeds as a function of traffic volume and lane capacity (Table S3), and estimates of vehicle-miles traveled by urban area and year (Table S4). Click here for file
