# Supplementary material for: Evaluation of the public health impacts of traffic congestion: a health risk assessment
Source: Environ Health. 2010 Oct 27;9:65. doi: 10.1186/1476-069X-9-65 (PMC2987789; doi:10.1186/1476-069X-9-65)
Supplement: Additional file 1 — Supplemental information. The supplemental information file includes a list of the 83 urban areas included in the model (Table S1), methods for estimating the percent of travel time in congestion (Table S2), estimates of vehicle speeds as a function of traffic volume and lane capacity (Table S3), and estimates of vehicle-miles traveled by urban area and year (Table S4). [file 1476-069X-9-65-S1.DOC]

Table S1. 83 Modeled Urban Areas

| Akron, OH | Detroit, MI | Philadelphia, PA--NJ--DE--MD |
| --- | --- | --- |
| Albany, NY | El Paso, TX—NM | Phoenix--Mesa, AZ |
| Albuquerque, NM | Eugene, OR | Pittsburgh, PA |
| Allentown--Bethlehem, PA--NJ | Fresno, CA | Portland, OR--WA |
| Atlanta, GA | Grand Rapids, MI | Providence, RI--MA |
| Austin, TX | Hartford, CT | Raleigh, NC |
| Bakersfield, CA | Houston, TX | Richmond, VA |
| Baltimore, MD | Indianapolis, IN | Riverside--San Bernardino, CA |
| Beaumont, TX | Jacksonville, FL | Rochester, NY |
| Birmingham, AL | Kansas City, MO—KS | Sacramento, CA |
| Boston, MA--NH--RI | Laredo, TX | St. Louis, MO--IL |
| Boulder, CO | Las Vegas, NV | Salem, OR |
| Bridgeport--Stamford, CT--NY | Little Rock, AR | Salt Lake City, UT |
| Brownsville, TX | Los Angeles--Long Beach--Santa Ana, CA | San Antonio, TX |
| Buffalo, NY | Louisville, KY—IN | San Diego, CA |
| Cape Coral, FL | Memphis, TN--MS—AR | San Francisco--Oakland, CA |
| Charleston--North Charleston, SC | Miami, FL | San Jose, CA |
| Charlotte, NC--SC | Milwaukee, WI | Sarasota--Bradenton, FL |
| Chicago, IL--IN | Minneapolis--St. Paul, MN | Seattle, WA |
| Cincinnati, OH--KY--IN | Nashville-Davidson, TN | Spokane, WA--ID |
| Cleveland, OH | New Haven, CT | Springfield, MA--CT |
| Colorado Springs, CO | New Orleans, LA | Tampa--St. Petersburg, FL |
| Columbia, SC | New York--Newark, NY--NJ—CT | Toledo, OH--MI |
| Columbus, OH | Oklahoma City, OK | Tucson, AZ |
| Corpus Christi, TX | Omaha, NE—IA | Tulsa, OK |
| Dallas--Fort Worth--Arlington, TX | Orlando, FL | Virginia Beach, VA |
| Dayton, OH | Oxnard, CA | Washington, DC--VA--MD |
| Denver--Aurora, CO | Pensacola, FL—AL |  |

Table S2: Roadway Congestion Index Calculation

| RCI Range | Equation for % Travel in Congestion |
| --- | --- |
| < 0.725 | RCI*30.34 |
| 0.725 – 0.925 | 22 + ((0.925-RCI)*55) |
| 0.925 – 1.025 | 33 + ((1.025-RCI)*70 |
| 1.025 – 1.4 | 40 + ((1.4-RCI)*26.67) |
| ≥ 1.4 | 50 |

Table S3: Daily Traffic Volume per Lane and Speed Estimates

| Facility and  Congestion Level | Daily Traffic  Volume per Lane | Speed Estimate Equation^1^ | |
| --- | --- | --- | --- |
|  |  | Peak Direction | Off-Peak Direction |
| Freeway |  | Lowest speed is 35 mph | Lowest speed is 40 mph |
| Uncongested | Under 15,000 | 60 | 60 |
| Medium | 15,001-17,500 | 70-(0.9*ADT/Lane) | 67-(0.6*ADT/Lane) |
| Heavy | 17,501-20,000 | 78-(1.4*ADT/Lane) | 71-(0.85*ADT/Lane) |
| Severe | 20,001-25,000 | 96-(2.3*ADT/Lane) | 88-(1.7*ADT/Lane) |
| Extreme | Over 25,000 | 76-(1.46*ADT/Lane) | 85.7-(1.6*ADT/Lane) |
| Arterial Street |  | Lowest speed is 20 mph | Lowest speed is 27 mph |
| Uncongested | Under 5,500 | 35 | 35 |
| Medium | 5,501-7,000 | 33.58-(0.74*ADT/Lane) | 33.82-(0.59 *ADT/Lane) |
| Heavy | 7,001-8,500 | 33.80-(0.77*ADT/Lane) | 33.90-(0.59*ADT/Lane) |
| Severe | 8,501-10,000 | 31.65-(0.51*ADT/Lane) | 30.10-(0.15*ADT/Lane) |
| Extreme | Over 10,000 | 32.57-(0.62*ADT/Lane) | 31.23-(0.27*ADT/Lane) |

1 – ADT/Lane in Thousands (ADT = average daily traffic)

Table S4: Predicted Vehicle-Miles Traveled by Urban Area and Year, Sorted by Percent Increase from 2005 to 2030.

| Urban Area | 2005 | 2010 | 2015 | 2020 | 2025 | 2030 | Difference |
| --- | --- | --- | --- | --- | --- | --- | --- |
| Raleigh, NC | 5.31E+06 | 7.54E+06 | 9.26E+06 | 1.07E+07 | 1.23E+07 | 1.41E+07 | 45% |
| Oxnard, CA | 1.76E+06 | 2.15E+06 | 2.63E+06 | 3.25E+06 | 3.87E+06 | 4.36E+06 | 42% |
| Richmond, VA | 4.95E+06 | 6.00E+06 | 7.16E+06 | 8.52E+06 | 1.02E+07 | 1.16E+07 | 40% |
| San Antonio, TX | 5.79E+06 | 7.21E+06 | 8.86E+06 | 1.07E+07 | 1.21E+07 | 1.35E+07 | 40% |
| Seattle, WA | 8.37E+05 | 1.01E+06 | 1.20E+06 | 1.42E+06 | 1.65E+06 | 1.92E+06 | 39% |
| San Diego, CA | 8.10E+06 | 9.89E+06 | 1.15E+07 | 1.32E+07 | 1.55E+07 | 1.80E+07 | 38% |
| Pensacola, FL--AL | 2.17E+06 | 2.74E+06 | 3.22E+06 | 3.73E+06 | 4.29E+06 | 4.79E+06 | 38% |
| Columbia, SC | 2.39E+06 | 2.88E+06 | 3.37E+06 | 3.96E+06 | 4.67E+06 | 5.22E+06 | 37% |
| Orlando, FL | 1.15E+07 | 1.46E+07 | 1.78E+07 | 2.00E+07 | 2.22E+07 | 2.45E+07 | 36% |
| Nashville-Davidson, TN | 4.00E+06 | 4.73E+06 | 5.46E+06 | 6.26E+06 | 7.15E+06 | 8.26E+06 | 35% |
| Kansas City, MO--KS | 7.86E+06 | 9.17E+06 | 1.06E+07 | 1.21E+07 | 1.39E+07 | 1.62E+07 | 35% |
| Las Vegas, NV | 1.08E+07 | 1.34E+07 | 1.55E+07 | 1.76E+07 | 1.98E+07 | 2.21E+07 | 34% |
| Cape Coral, FL | 3.48E+06 | 4.41E+06 | 4.94E+06 | 5.46E+06 | 6.00E+06 | 6.56E+06 | 31% |
| Laredo, TX | 2.24E+06 | 2.63E+06 | 3.02E+06 | 3.42E+06 | 3.82E+06 | 4.23E+06 | 31% |
| San Francisco--Oakland, CA | 2.01E+07 | 2.38E+07 | 2.67E+07 | 2.98E+07 | 3.31E+07 | 3.69E+07 | 30% |
| Tulsa, OK | 3.70E+06 | 4.23E+06 | 4.76E+06 | 5.34E+06 | 5.99E+06 | 6.79E+06 | 30% |
| Colorado Springs, CO | 4.05E+06 | 4.79E+06 | 5.37E+06 | 5.99E+06 | 6.67E+06 | 7.42E+06 | 29% |
| Charleston--North Charleston, SC | 3.86E+06 | 4.53E+06 | 5.26E+06 | 6.03E+06 | 6.51E+06 | 7.02E+06 | 29% |
| Springfield, MA--CT | 9.77E+05 | 1.11E+06 | 1.25E+06 | 1.41E+06 | 1.57E+06 | 1.76E+06 | 29% |
| Corpus Christi, TX | 1.85E+06 | 2.08E+06 | 2.34E+06 | 2.66E+06 | 3.03E+06 | 3.34E+06 | 29% |
| Grand Rapids, MI | 2.70E+06 | 3.02E+06 | 3.39E+06 | 3.79E+06 | 4.24E+06 | 4.76E+06 | 28% |
| Jacksonville, FL | 6.46E+06 | 7.75E+06 | 8.54E+06 | 9.34E+06 | 1.02E+07 | 1.12E+07 | 27% |
| Albuquerque, NM | 4.90E+06 | 5.61E+06 | 6.23E+06 | 6.90E+06 | 7.63E+06 | 8.45E+06 | 27% |
| Phoenix--Mesa, AZ | 3.32E+07 | 3.79E+07 | 4.22E+07 | 4.65E+07 | 5.08E+07 | 5.54E+07 | 25% |
| Salt Lake City, UT | 3.94E+05 | 4.45E+05 | 4.90E+05 | 5.39E+05 | 5.93E+05 | 6.54E+05 | 25% |
| Tucson, AZ | 6.40E+06 | 7.45E+06 | 8.49E+06 | 9.25E+06 | 9.91E+06 | 1.06E+07 | 25% |
| Bakersfield, CA | 3.86E+06 | 4.45E+06 | 4.95E+06 | 5.50E+06 | 6.01E+06 | 6.38E+06 | 25% |
| Charlotte, NC--SC | 1.12E+07 | 1.33E+07 | 1.45E+07 | 1.58E+07 | 1.71E+07 | 1.84E+07 | 24% |
| Austin, TX | 1.12E+07 | 1.27E+07 | 1.40E+07 | 1.53E+07 | 1.66E+07 | 1.80E+07 | 23% |
| Riverside--San Bernardino, CA | 1.86E+07 | 2.09E+07 | 2.31E+07 | 2.52E+07 | 2.74E+07 | 2.97E+07 | 23% |
| Fresno, CA | 5.57E+06 | 6.30E+06 | 7.05E+06 | 7.80E+06 | 8.29E+06 | 8.82E+06 | 23% |
| Miami, FL | 5.62E+07 | 6.18E+07 | 6.81E+07 | 7.46E+07 | 8.12E+07 | 8.80E+07 | 22% |
| Omaha, NE--IA | 4.95E+06 | 5.44E+06 | 5.92E+06 | 6.45E+06 | 7.03E+06 | 7.68E+06 | 22% |
| Eugene, OR | 1.99E+06 | 2.22E+06 | 2.47E+06 | 2.69E+06 | 2.85E+06 | 3.03E+06 | 21% |
| Atlanta, GA | 4.03E+07 | 4.66E+07 | 5.09E+07 | 5.42E+07 | 5.76E+07 | 6.11E+07 | 21% |
| Boulder, CO | 1.14E+06 | 1.28E+06 | 1.43E+06 | 1.52E+06 | 1.62E+06 | 1.72E+06 | 20% |
| Minneapolis--St. Paul, MN | 2.27E+07 | 2.48E+07 | 2.73E+07 | 2.99E+07 | 3.21E+07 | 3.39E+07 | 20% |
| Oklahoma City, OK | 8.29E+06 | 9.41E+06 | 1.01E+07 | 1.08E+07 | 1.15E+07 | 1.24E+07 | 20% |
| Sacramento, CA | 1.62E+07 | 1.76E+07 | 1.92E+07 | 2.08E+07 | 2.24E+07 | 2.41E+07 | 20% |
| El Paso, TX--NM | 7.88E+06 | 8.63E+06 | 9.36E+06 | 1.01E+07 | 1.09E+07 | 1.17E+07 | 19% |
| Allentown--Bethlehem, PA--NJ | 3.90E+06 | 4.42E+06 | 4.75E+06 | 5.05E+06 | 5.39E+06 | 5.77E+06 | 19% |
| Baltimore, MD | 1.83E+07 | 1.97E+07 | 2.15E+07 | 2.34E+07 | 2.55E+07 | 2.71E+07 | 19% |
| Dallas--Fort Worth--Arlington, TX | 5.04E+07 | 5.79E+07 | 6.19E+07 | 6.59E+07 | 7.00E+07 | 7.44E+07 | 19% |
| Denver--Aurora, CO | 2.26E+07 | 2.58E+07 | 2.75E+07 | 2.93E+07 | 3.12E+07 | 3.32E+07 | 19% |
| Columbus, OH | 9.90E+06 | 1.07E+07 | 1.15E+07 | 1.24E+07 | 1.33E+07 | 1.44E+07 | 19% |
| Brownsville, TX | 2.01E+06 | 2.19E+06 | 2.35E+06 | 2.52E+06 | 2.69E+06 | 2.87E+06 | 18% |
| Indianapolis, IN | 1.04E+07 | 1.13E+07 | 1.21E+07 | 1.30E+07 | 1.40E+07 | 1.48E+07 | 18% |
| Virginia Beach, VA | 1.45E+07 | 1.54E+07 | 1.67E+07 | 1.80E+07 | 1.95E+07 | 2.06E+07 | 18% |
| Portland, OR--WA | 1.70E+07 | 1.82E+07 | 1.94E+07 | 2.06E+07 | 2.18E+07 | 2.31E+07 | 15% |
| Houston, TX | 4.90E+07 | 5.31E+07 | 5.61E+07 | 5.92E+07 | 6.24E+07 | 6.58E+07 | 15% |
| Providence, RI--MA | 1.07E+07 | 1.11E+07 | 1.18E+07 | 1.26E+07 | 1.34E+07 | 1.43E+07 | 15% |
| Little Rock, AR | 1.39E+06 | 1.48E+06 | 1.56E+06 | 1.64E+06 | 1.74E+06 | 1.86E+06 | 14% |
| Spokane, WA--ID | 2.73E+07 | 2.91E+07 | 3.08E+07 | 3.25E+07 | 3.44E+07 | 3.63E+07 | 14% |
| Tampa--St. Petersburg, FL | 2.24E+07 | 2.39E+07 | 2.53E+07 | 2.67E+07 | 2.81E+07 | 2.97E+07 | 14% |
| Birmingham, AL | 7.02E+06 | 7.41E+06 | 7.82E+06 | 8.26E+06 | 8.75E+06 | 9.31E+06 | 14% |
| New Haven, CT | 3.96E+06 | 4.17E+06 | 4.43E+06 | 4.72E+06 | 4.97E+06 | 5.21E+06 | 14% |
| Beaumont, TX | 3.46E+05 | 3.51E+05 | 3.68E+05 | 3.87E+05 | 4.09E+05 | 4.34E+05 | 11% |
| Memphis, TN--MS--AR | 1.04E+07 | 1.09E+07 | 1.13E+07 | 1.18E+07 | 1.23E+07 | 1.30E+07 | 11% |
| Washington, DC--VA--MD | 4.22E+07 | 4.40E+07 | 4.59E+07 | 4.78E+07 | 4.98E+07 | 5.20E+07 | 10% |
| Louisville, KY--IN | 1.03E+07 | 1.07E+07 | 1.11E+07 | 1.16E+07 | 1.21E+07 | 1.26E+07 | 10% |
| Chicago, IL--IN | 9.75E+07 | 1.01E+08 | 1.04E+08 | 1.08E+08 | 1.12E+08 | 1.17E+08 | 9% |
| Pittsburgh, PA | 7.81E+06 | 7.88E+06 | 8.14E+06 | 8.46E+06 | 8.85E+06 | 9.34E+06 | 9% |
| Boston, MA--NH--RI | 3.01E+07 | 3.09E+07 | 3.17E+07 | 3.27E+07 | 3.39E+07 | 3.53E+07 | 8% |
| Los Angeles--Long Beach--Santa Ana, CA | 1.32E+08 | 1.36E+08 | 1.40E+08 | 1.44E+08 | 1.49E+08 | 1.54E+08 | 8% |
| Hartford, CT | 7.03E+06 | 7.23E+06 | 7.43E+06 | 7.68E+06 | 7.93E+06 | 8.18E+06 | 8% |
| Milwaukee, WI | 1.21E+07 | 1.24E+07 | 1.27E+07 | 1.31E+07 | 1.35E+07 | 1.40E+07 | 7% |
| Cincinnati, OH--KY--IN | 1.37E+07 | 1.40E+07 | 1.44E+07 | 1.47E+07 | 1.52E+07 | 1.58E+07 | 7% |
| Philadelphia, PA--NJ--DE--MD | 5.06E+07 | 5.24E+07 | 5.36E+07 | 5.49E+07 | 5.63E+07 | 5.80E+07 | 7% |
| New York--Newark, NY--NJ--CT | 1.72E+08 | 1.75E+08 | 1.80E+08 | 1.85E+08 | 1.90E+08 | 1.96E+08 | 7% |
| San Jose, CA | 3.13E+07 | 3.21E+07 | 3.29E+07 | 3.37E+07 | 3.47E+07 | 3.57E+07 | 7% |
| Bridgeport--Stamford, CT--NY | 9.60E+06 | 9.89E+06 | 1.01E+07 | 1.04E+07 | 1.06E+07 | 1.09E+07 | 7% |
| Sarasota--Bradenton, FL | 1.65E+07 | 1.70E+07 | 1.73E+07 | 1.77E+07 | 1.81E+07 | 1.85E+07 | 6% |
| Akron, OH | 4.84E+06 | 4.90E+06 | 4.99E+06 | 5.09E+06 | 5.22E+06 | 5.38E+06 | 5% |
| St. Louis, MO--IL | 6.67E+05 | 6.75E+05 | 6.85E+05 | 6.99E+05 | 7.17E+05 | 7.40E+05 | 5% |
| Albany, NY | 2.23E+06 | 2.26E+06 | 2.27E+06 | 2.30E+06 | 2.35E+06 | 2.42E+06 | 4% |
| Buffalo, NY | 5.07E+06 | 5.01E+06 | 5.05E+06 | 5.12E+06 | 5.23E+06 | 5.40E+06 | 3% |
| Rochester, NY | 4.51E+06 | 4.50E+06 | 4.52E+06 | 4.57E+06 | 4.66E+06 | 4.80E+06 | 3% |
| Toledo, OH--MI | 4.02E+06 | 3.99E+06 | 4.01E+06 | 4.06E+06 | 4.14E+06 | 4.26E+06 | 3% |
| Detroit, MI | 4.05E+07 | 4.04E+07 | 4.07E+07 | 4.12E+07 | 4.19E+07 | 4.29E+07 | 3% |
| Salem, OR | 2.25E+07 | 2.26E+07 | 2.27E+07 | 2.29E+07 | 2.31E+07 | 2.34E+07 | 2% |
| Dayton, OH | 4.91E+06 | 4.86E+06 | 4.86E+06 | 4.90E+06 | 4.97E+06 | 5.11E+06 | 2% |
| New Orleans, LA | 7.78E+06 | 3.91E+06 | 4.95E+06 | 6.12E+06 | 6.97E+06 | 7.92E+06 | 1% |
| Cleveland, OH | 1.49E+07 | 1.44E+07 | 1.41E+07 | 1.39E+07 | 1.36E+07 | 1.34E+07 | -5% |
